# Yeast Viability in HLD–NAC-Designed Fully Dilutable Lecithin-Linker Microemulsions

**DOI:** 10.3390/molecules30040921

**Published:** 2025-02-17

**Authors:** Juan Doratt Mendoza, Jingwen Ding, Michelle Acosta Alvarez, Edgar Acosta

**Affiliations:** Department of Chemical Engineering and Applied Chemistry, Faculty of Engineering and Applied Science, University of Toronto, Toronto, ON M5S 3E5, Canada; juan.doratt@mail.utoronto.ca (J.D.M.); jw.ding@alumni.utoronto.ca (J.D.);

**Keywords:** yeast, microemulsion, lecithin, linkers, HLD–NAC, biologics

## Abstract

Using microemulsions (µEs) as preservation media for cells was pursued in the 1990s; however, the difficulty in formulating biocompatible µEs and keeping unacclimatized cells alive for more than three days hindered developments in this area. This work explores the use of fully dilutable self-microemulsifying delivery systems (SMEDS) formulated with lecithin (Le) and polyglycerol-10-caprylate (PG10C) at a ratio of 2/5. This surfactant blend was mixed with ethyl oleate (EOL) at a ratio of 60 surfactant/40 EOL to produce a D60 dilution line. This D60 SMEDS was diluted with 0.9% *w*/*v* NaCl solution to produce lecithin-linker µEs (LLMs). The properties of the resulting LLMs were predicted using the hydrophilic–lipophilic-difference (HLD) and net-average curvature (NAC) model, indicating that LLMs with aqueous content from 5% to 60% are bicontinuous, confirmed via viscosity and conductivity. The largest yeast activity and viability obtained with LLMs were achieved with 30% aqueous content, resulting from the balance between having enough water for the effective transport of metabolites, enough SMEDS to contribute nutrients and lipids, and a low enough water to limit the partition of PG10C that, when present in the aqueous phase, inhibited yeast activity. For SMEDS, its low water activity ensured that the yeast remained dormant, keeping them alive for at least 10 weeks.

## 1. Introduction

Microemulsions (µEs) are surfactant–oil–water (SOW) systems that exist in thermodynamic equilibrium. µEs contain hydrophobic and hydrophilic compounds that could contribute to or hinder microorganism viability and activity. As a result, µEs could be used as a fermentation media, a disinfecting media, and possibly as encapsulating media/delivery system for cells. The main difficulty in formulating for any of these applications is understanding how the selection of surfactants and the proportion of the surfactant, oil, and water in the system can affect the viability and activity of microorganisms. This is the main question explored in this work using yeast as a model microorganism.

Luisi’s group was one of the first to evaluate the activity of yeast in water-in-oil µEs formulated in three different SOW systems: (a) PEG-20-sorbitan trioleate (Tween 85) as surfactant and isopropyl palmitate (IPP) as oil, (b) azolectin (a form of lecithin) and IPP, (c) azolectin and hexadecane [1]. Luisi’s group used yeast (*Saccharomyces cerevisiae*) because of its biotechnological importance, its relatively large size that facilitates the use of microscopy to evaluate the impact of µEs on viability, and its relative fragility. The researchers found that, when holding the water/surfactant molar ratio (Wo) to 14, the original yeast has a greater viability in the formulation with azolectin and IPP and can survive for 3 days in that formulation. The authors determined that cells acclimatized to the µE environment could survive for 10 days. Hoppert et al. evaluated the photosynthetic activity of cyanobacteria and microalgae cells in water-in-oil µEs formulated with Tween 85 and sorbitan monooleate (Span 80) as surfactants and hexadecane as the oil phase [2]. In those experiments, the authors found that maximum photoactivity was obtained when Wo ranged from 80 to 180. The authors further proposed that in water-in-oil µEs, microorganisms are surrounded by a thin film of water with properties that are different from bulk properties but similar to the behavior of water in the confined environment of cells. The Luisi group also used the Tween 85-Span 80-hexadecane system as a suspension media for *Candida pseudotropicalis* and determined that these µEs, with Wo~10 to 20, can disperse the cells and promote anabolic and catabolic activity that used carbon from hexadecane (based on radiolabeled hexadecane) as a carbon source [3]. Prichanon et al. also used the Tween 85 + Span 80—hexadecane system to suspend *Mycobacterium* sp. [4]. In their work, the authors determined that higher water contents reaching Wo~25 could lead to better dispersion of the cells. Although their article refers to this dispersion of cells in µEs as an encapsulation process, the authors acknowledge that their main concern is the dispersion of the cells and not evaluating their survival or activity. In a follow-up article, the authors use these µEs as a way to suspend the heat-killed cells of *Mycobacterium* sp. where the released enzymes from the cells were capable of catalyzing epoxidation reactions [5].

The momentum of the research on microorganism viability in µEs was lost after the early 2000s, due to the limited viability of the cells after a few days of exposure to the µEs and the complexity of formulating biocompatible µEs with a wide range of Wo ratios. Furthermore, even if the cell remains alive, metabolic and anabolic processes will keep changing the composition of the µEs, leading to changes in the thermodynamic state of the µE, which can result in phase separation. At that point, the attention concentrated on hosting microbial cells in water-in-oil macro- or regular emulsions [6], on the evaluation of enzymatic activity in µEs, and the use of µEs (containing antimicrobial components) as antimicrobial formulations [7].

Much has changed since the early 2000s, including the development of the biocompatible lecithin-linker µEs (LLMs) [8]. There are two types of linkers: lipophilic linkers (LL, long-chain, C12+ amphiphiles with weakly hydrophilic headgroups such as alcohols, monoglycerols, and fatty acids) and hydrophilic linkers (HL, C6–C10 surfactant molecules with highly hydrophilic headgroups such as polyglycerols, polyethylene glycols, etc.). The advantage of using linkers is that it reduces the number of surfactants and co-surfactants in the formulation and allows for a broader selection of additives that can be biocompatible. Using lecithin (Le) as a surfactant, C8–C10 polyglycerols as HL, and glycerol monooleate as LL, Nouraei et al. formulated self-microemulsifying delivery systems (SMEDS) capable of increasing the intestinal absorption of ibuprofen by threefold [9]. SMEDS are water-free LLMs; in other words, SMEDS are surfactant–oil (SO) systems that, when diluted with water, form single-phase µEs. Fully dilutable SMEDS can provide the entire range of water to the surfactant mol ratio (Wo~ from 0 to 1000s), which was not possible when previous cell viability studies in µEs were conducted. Furthermore, although the work of Luisi’s group pointed to azolectin (a form of lecithin) as the best surfactant, most of the other studies concentrated on polyethylene glycol (PEG) surfactants like Tween 85 because they are easier to formulate with those surfactants than with lecithin. The hydrophilic linker (HL), in particular, reduces the interfacial rigidity of lecithin membranes, allowing the formulation of lecithin µEs with a wide range of oils and aqueous solutions [8,10,11].

Nouraei et al. [9] also introduced the use of the hydrophilic–lipophilic difference and net-average curvature (HLD–NAC) model to predict the minimum surfactant (Le + HL + LL) to oil ratio required to produce SMEDS. The HLD model for non-ionic surfactants, such as the ones used in Nouraei et al. [9], is shown below:

HLD = b∙S − k∙EACN + c_T_(T − 25 °C) + Cc (1)

The HLD indicates the approach to the phase inversion point (PIP) of emulsions and µEs. For SOW systems (emulsions and saturated µEs) with a water-to-oil ratio (WOR) between 1/3 and 3/1, negative HLDs indicate that water tends to be in the continuous phase. Positive HLDs correspond to SOW systems where the oil tends to be in the continuous phase. As the SOW system approaches HLD~0, the system tends to produce bicontinuous phases. The term b∙S accounts for the salinity (S) of the system, measured in grams of NaCl per 100 mL, where b~0.13. The parameter “k” depends on the surfactant, but, for lecithin and linkers, k~0.16. EACN is the equivalent alkane carbon number of the oil; for n-alkanes between hexane and hexadecane, this is simply the number of carbon atoms in the chain, but, for any other oil, this is obtained from salinity scans [12]. The term c_T_ (T-25 °C) reflects the temperature dependence and, for PEG-based non-ionic surfactants, an increase in temperature results in a more hydrophobic SOW system (c_T_~0.06 °C^−1^). However, for lecithin-linker µEs that are PEG-free, these systems are less temperature-dependent (c_T_~0.01 °C^−1^) [8]. The term Cc in Equation (1) represents the surfactant’s characteristic curvature value, indicating its inherent hydrophobicity. Surfactants that are more hydrophobic have more positive Cc values.

While the HLD is very useful, it does not explain the effect of surfactant concentration and does not, on its own, predict water or oil solubilization capacity or the µE phase boundary of ternary phase diagrams (TPDs). The NAC model, combined with the HLD, can predict these solubilization capacities and the single-phase boundary of TPDs, even for lecithin-linker µEs [13]. The NAC utilizes a mathematical model of two coexisting states to describe SOW systems. In one state, the aqueous phase is continuous where the oil solubilized has a solubilization radius Ro, and, in the other state, the oil phase is continuous where the aqueous phase solubilized has a solubilization radius Rw. This dual-state approach defines the net (Hn) and average (Ha) curvatures as follows:

(2) $$H_{n}=\frac{1}{R_{o}}-\frac{1}{R_{w}}=\left(\frac{\phi_{s}}{3\phi_{o}}-\frac{\phi_{s}}{3\phi_{w}}\right)\left(\frac{a_{s}}{v_{s}}\right)=-\frac{\mathrm{HLD}}{L}$$

(3) $$H_{a}=\frac{1}{2}\left(\frac{1}{R_{o}}+\frac{1}{R_{w}}\right)=\frac{1}{2}\left(\frac{\phi_{s}}{3\phi_{o}}+\frac{\phi_{s}}{3\phi_{w}}\right)\left(\frac{a_{s}}{v_{s}}\right)\geq \frac{1}{\xi }$$

where ϕ_o_, ϕ_w_, ϕ_s_ are the volume fractions of oil, water, and surfactant in the SOW system. The ratio a_s_/v_s_ is the interfacial area to molecular volume ratio of the surfactant. The surfactant tail length parameter, L, is fitted using experimental solubilization, but, for most surfactants, it is approximately 1.2 to 1.4 times the surfactant tail length [14]. The parameter ξ is the µE characteristic length, which indicates the maximum oil and water solubilization capacity of the SOW system. The combined HLD–NAC model is now used as an equation of state for µEs that can be used to define the optimal formulations for a given surfactant and oil composition.

Given the recent advances in fully dilutable, lecithin-linker SMEDS approach, which produces lecithin-linker µEs (LLM) upon dilution with aqueous solutions, and the HLD–NAC model to design these SMEDS; the question explored in this work is, can SMEDS or their resulting LLMs maintain unacclimatized yeast alive and active beyond the 3 days reported in previous studies? We hypothesize that *Saccharomyces cerevisiae* will survive in the different microenvironments (reverse micellar, bicontinuous, and micellar systems) of LLMs). Confirmation of this hypothesis could open the doors for SMEDS and LLMs as delivery media for cells in applications such as probiotics delivery, for LLMs as solvent media in bioreactors for pharmaceutical applications, and for LLMs as adjuvants in remediation applications, such as oil spills [15].

To explore this hypothesis, we formulated LLMs using polyglyceryl-10 caprylate (PG10C, a HL), soy lecithin (Le, surfactant), and ethyl oleate (EOL, oil). The characteristic curvature (Cc) of Le and PG10C were obtained using surfactant µE phase scans using a C9E5 alkyl ethoxylated surfactant as a reference nonionic surfactant [12]. The EACN of EOL was obtained using a salinity µE phase scan using a C9E5 alkyl ethoxylated surfactant as a reference nonionic surfactant. A surfactant composition scan with Le, PG10C and EOL was then conducted and fitted using the HLD–NAC model to obtain the NAC parameters (L, ξ). These parameters were used to predict a TPD that could produce fully dilutable formulations using the lowest possible surfactant-to-oil ratio. The viability and activity of *Saccharomyces cerevisiae* along the fully dilutable line of the selected formulation were assessed via methylene blue cell staining and CO_2_ production tests, respectively. The electrical conductivity and viscosity of µEs obtained along the dilution line were evaluated as a function of water content (and Wo ratio) to evaluate the microenvironment of each µE, considering the HLD–NAC predicted µE morphology.

## 2. Results and Discussions

### 2.1. Determination of HLD Parameters

**Figure 1:**
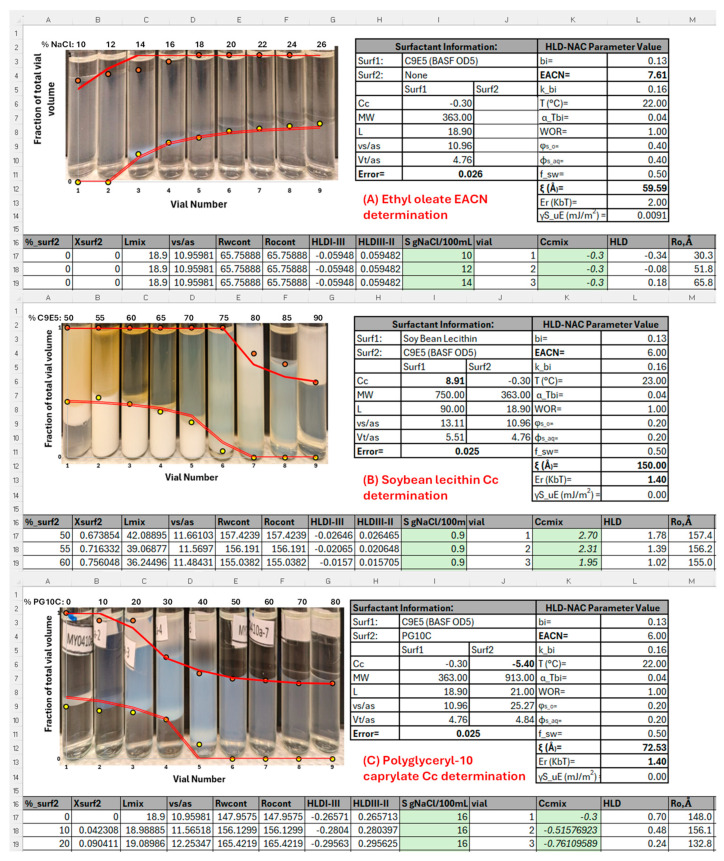
(**A**) Salinity scan used to obtain the Equivalent Alkane Carbon Number (EACN) of ethyl oleate (oil) using pentaethylene glycol nonyl ether (C9E5 or OD5, surfactant 1); the numbers on top of each vial represent the %*w*/*v* NaCl (salinity) in the aqueous phase. (**B**) Surfactant composition scan with lecithin (SBlec, surfactant 1) and reference surfactant C9E5 (surfactant 2) using limonene (EACN = 6) as oil and a 0.9% *w*/*v* NaCl aqueous phase; the numbers on top of each vial represent the wt% of C9E5 (surf2) in the mixture with lecithin (wt%surf1 = 100 − wt%surf2). (**C**) Surfactant composition scan with reference surfactant C9E5 (surfactant 1) and polyglycerol-10 caprylate (PG10C, surfactant 2) using limonene (EACN = 6) as oil and a 0.9% *w*/*v* NaCl in the aqueous phase; the numbers on top of each vial represent the wt% of PG10C (surf2) in the mixture with C9E5 (surf1). The orange and yellow dots are the experimental determination of the lower level (LL) and upper level (UL) boundaries of the µE phases. The solid red lines are predictions of the lower level (LL) and upper level (UL) boundaries of the µE phases predicted by the HLD–NAC programmed in the HLD–NAC tutorial [16], using the algorithm explained in Figure 2.

Figure 1A presents a partial screenshot of the HLD–NAC spreadsheet used to fit the salinity scan for the equivalent alkane carbon number (EACN) of ethyl oleate (EOL), the oil used to prepare the lecithin-linker µEs (LLMs). The spreadsheet used was a modified version of the published HLD–NAC tutorial spreadsheet [16], which was designed for the salinity scan of a single ionic surfactant16. The salinities in column “I” (S = 10, 12, …, 26% NaCl) were used to calculate the HLD in column “L” using Equation (1). The HLD–NAC parameters for the OD5 reference surfactant are shown in cells B12 through B15. The HLD–NAC-predicted lower and upper µE levels (LL, UL) are included in columns “O” and “P”. Cell I11 calculates the error between the predicted LL and UL and the experimental levels (red circles, UL; yellow circles LL). The fitted parameters were the EACN of EOL and the characteristic length (ξ) of the µE. The EACN = 7.6 obtained in Figure 1A is close to the EACN = 7.3 for EOL reported by Ontiveros and collaborators [17].

**Figure 2:**
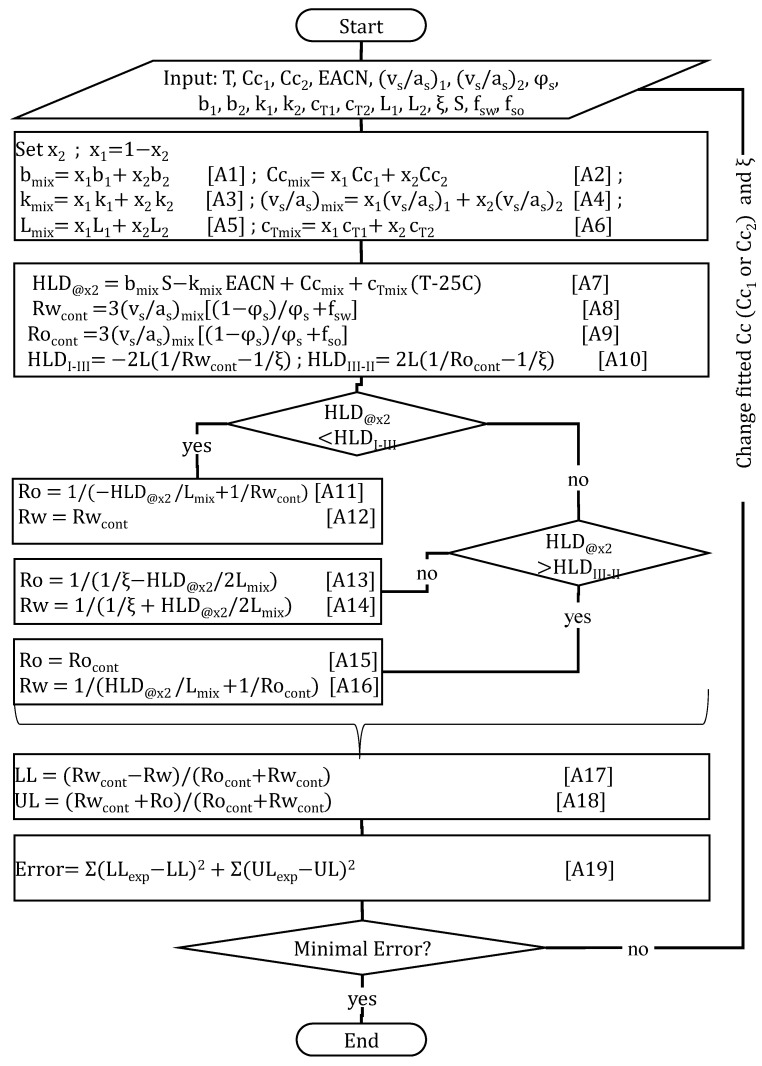
HLD–NAC algorithm to calculate the lower level (LL) and upper level (UL) of µEs produced in composition (x_2_) phase scans at water/oil volume ratio (WOR) of 1/1.

Figure 1B presents a partial screenshot of the HLD–NAC spreadsheet used to fit the surfactant composition scan for the characteristic curvature (Cc) of lecithin (Le, surfactant 1) mixed with OD5 (surfactant 2), maintaining S = 0.9% *w*/*v* NaCl (column “I”), and using limonene (EACN = 6) as the oil phase. The percentage of OD5 (in the mixture with Le) was gradually increased from 50 wt% to 90 wt% in 5% intervals (cells A17 to A25). For each composition, the molar fraction of surfactant 2 (xsurf2, cells B17 to B25) was calculated considering the molecular weights (MW, cells I7, J7) of each surfactant. The Cc of the mixture was then calculated in cells K17 to K25 using the linear mixing rule. The HLD of the SOW system was calculated using Equation (1) and Cc_mix_. The parameters fitted in this case were the Cc of lecithin (cell I6) and the characteristic length (ξ, cell L12). The complete set of equations are included in the algorithm of Figure 2. The fitted Cc of +8.9 is higher than the published value of +5.5 [13]. Unpublished studies in our group show that the Cc of lecithin can range from +4 to +9 depending on the lecithin source, something common for a product derived from natural sources. Figure 1C presents a similar study for PG10C, producing a Cc of −5.4, which is slightly less negative than Cc = −7.4, obtained in a previous study using 10% OD5 as a reference surfactant [15]. One important difference is that the 20% surfactant used in the current work tends to produce more accurate Cc values [18].

Figure 2 presents the algorithm used to generate the µE boundary (red) lines for composition (x2 or x_surf2_) phase scans. The algorithm starts by setting the temperature (T), salinity (S, g NaCl/100 mL), the HLD–NAC parameters for surfactants 1 and 2: their characteristic curvature (Cc_1_, Cc_2_), volume to area ratio ((vs/as)_1_, (vs/as)_2_), salinity factors (b_1_, b_2_), EACN factors (k_1_, k_2_), temperature factors (c_T1_, c_T2_), tail lengths (L_1_, L_2_), and the total surfactant volume fraction added to the aqueous phase (φ_s_), the µE characteristic length (ξ), and the fraction of the surfactant volume that contributes to the aqueous (f_sw_) and oil (f_so_) volume compartments.

**Figure 3:**
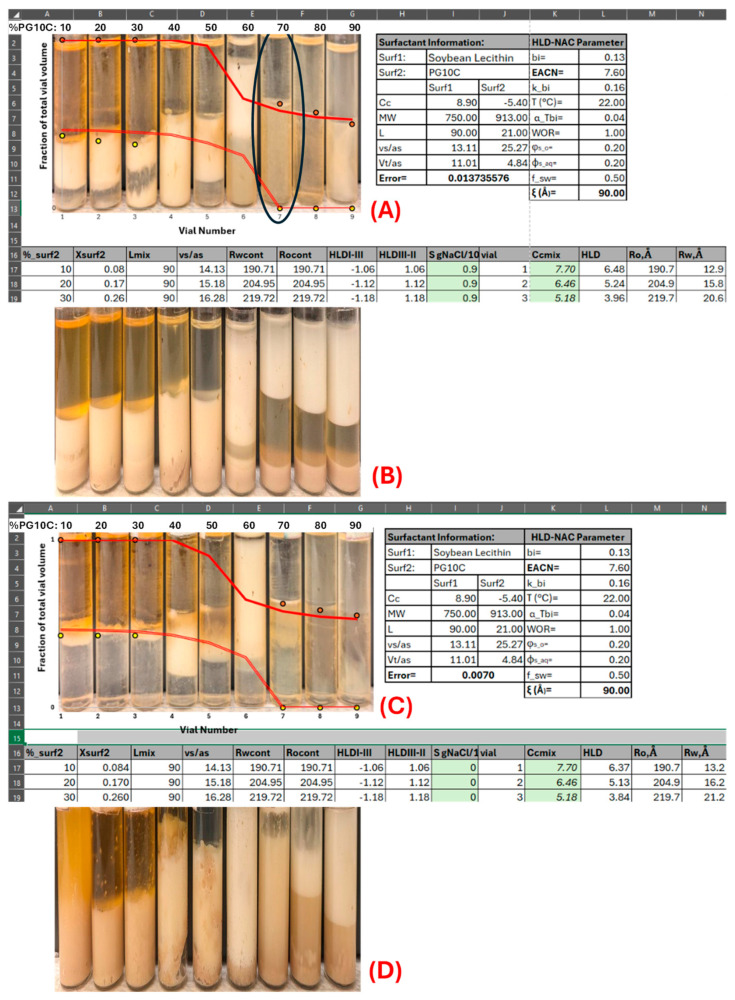
(**A**) Surfactant composition scan using mixtures of lecithin (surfactant 1) and PG10C (surfactant 2) in an aqueous phase containing 0.9% *w*/*v* NaCl. (**B**) Surfactant composition scan of panel A with 5 wt% yeast in aqueous phase. (**C**) Surfactant composition scan using mixtures of lecithin (surfactant 1) and PG10C (surfactant 2) in an aqueous phase containing 0.9% *w*/*v* NaCl and 25% *w*/*v* sucrose. (**D**) Surfactant composition scan of panel C with 5 wt% yeast in aqueous phase. The numbers on top of each vial represent the wt% of PG10C (surf2) in the mixture with lecithin (wt%surf1 = 100 − wt%surf2).

Equations (A1) through (A6) in Figure 2 are used to obtain the surfactant parameters for the mixture with a given molar fraction of surfactants 1 and 2 (x_1_, x_2_). Equation (A7) calculates the HLD of the surfactant mixture with composition x_2_. Equations (A8) and (A9) present the solubilization radii for the continuous aqueous and oil phases (Rw_cont_, Ro_cont_, respectively). Equation (A10) is used to calculate the HLD_I–III_ at the transition from Type I (water-continuous) to Type III (bicontinuous middle phase) µEs, and the HLD_III–II_ from Type III to Type II (oil-continuous) µEs. For Type I µEs, Equations (A11) and (A12) calculate the solubilization radii of oil (Ro) and water (Rw), respectively. For Type III µEs, Equations (A13) and (A14) calculate the solubilization radii of oil (Ro) and water (Rw), respectively. For Type II µEs, Equations (A15) and (A16) calculate the solubilization radii of oil (Ro) and water (Rw), respectively. These solubilization radii are then used in Equations (A17) and (A18) to calculate the lower (LL) and upper (UL) limits of the µE, expressed as volume fractions of the total volume of the SOW system. Equation (A19) calculates the deviation (error) between the experimental µE boundaries (LL_exp_, UL_exp_) and the HLD–NAC predicted LL and UL. To minimize the error, the fitted parameters (Cc_1_ or Cc_2_, and ξ) are adjusted. The input parameters and the fitted values in the composition scans of Figure 1B,C are shown in the screenshots presented in those figures. For the composition scans in Figure 3A,C, the L_mix_ was not calculated from composition but set to 90 Å, reflecting the synergism of lecithin-linker scans [9,13]. For the systems of Figure 3A,C the characteristic length was set to ξ = 90 Å.

### 2.2. Lecithin–Hydrophilic Linker (Le–HL) Phase Scans

Phase behavior studies of mixtures of lecithin (Le) and hydrophilic linker (HL, PG10C) were conducted to determine the lecithin-to-hydrophilic linker ratio required to produce water-dilutable µEs with the maximum possible oil solubilization. To estimate the HLD–NAC parameters L and ξ for lecithin-linker µEs, the Le-PG10C composition scan shown in Figure 3A was performed using EOL as the oil phase. The inversion points from water-in-oil µEs (vials 1 through 5 in Figure 3A) to oil-in-water µEs (vials 7 through 9) predicted by the HLD–NAC model was set by the EACN and Cc parameters obtained in Figure 1. The parameters L and ξ only help to fit the water or oil solubilization capacity of the µEs. The value of L = 90 Å is consistent with the value of L obtained from other lecithin-linker µEs [9,13]. The relatively low value of ξ = 90 Å compared to previous Le–LL–HL µEs (with ξ~200 Å) is likely due to the lack of a lipophilic linker (LL) [13].

Vials 1 through 6 in Figure 3A still display metastable emulsions (white phases) even after four heating cycles and 6 weeks of settling. This behavior is typical of lecithin µEs, especially those with low HL (PG10C, in this case) content. The surfactant mixture of vial #7 in Figure 3A has 70 parts PG10C, 30 parts Le, and a total surfactant concentration of 20 wt%. This vial is highlighted with an oval in Figure 3A because this is the approximate composition selected for ternary phase diagrams. At this surfactant composition, we do not observe metastable emulsions; the µE is continuous in the aqueous phase, which suggests that it can be further diluted with the aqueous phase, and the amount of oil solubilization is the maximum.

The phase scan in Figure 3B is the same scan as Figure 3A, but with 5% yeast added to the aqueous phase. The picture was taken one day after yeast addition. Overall, the phase behavior did not seem to be impacted by the addition of yeast, as the yellow tinge of the oil phases in vials #1 through #5 in Figure 3B are similar to the corresponding vials in Figure 3A. This yellow tinge is indicative of the presence of lecithin and PG10C in the oil, which is otherwise colorless. The presence of the surfactant in the oil phase is a signature for water-in-oil µEs. Similarly, the yellow tinge in the aqueous phases of vials 7 through 9 in Figure 3A,B are indicative of oil-in-water µEs. In all the vials, there was evidence of some level of yeast activity even though no sucrose was added. This is consistent with previous observations that yeast can use some of the oil, and perhaps the surfactants, as carbon sources for aerobic respiration [3].

Figure 3C presents a surfactant composition scan similar to that of Figure 3A, except that the aqueous phase contains 25 wt% sucrose. Using the same HLD–NAC parameters used in Figure 3A, the HLD–NAC predicted phase volumes in Figure 3C largely agree with the experimental values, except that vial #5 developed a middle phase µE, and vial #7 presented what appears to be a second phase in the aqueous phase, but this phase turned into a single clear aqueous phase with mild mixing. Given that sucrose is not interfacially active, it is not a surprise that it did not substantially affect the phase behavior of LLMs. One interesting observation is that, despite the increase in aqueous phase viscosity caused by the addition of sucrose, the system in Figure 3C produced less stable emulsions than the system of Figure 3A, a phenomenon that is worth exploring in future studies and may be related to the hydration of the lecithin headgroups.

Figure 3D shows the same scan as Figure 3C after adding 5% yeast. Evidence of vigorous CO_2_ production started within 10 min of yeast addition in vials # 5, 6, and 7. Due to the relatively large amount of gas produced, the caps of each vial were left loose to prevent pressure buildup. The picture in Figure 3D was taken 24 h after introducing the yeast showing that fermentation took place in all the vials. There was evidence that, for vials # 4 through 9, the evolution of CO_2_ was large enough to produce a froth that carried a portion of the liquid out of the vials.

### 2.3. Ternary Phase Diagram (TPD)

**Figure 4:**
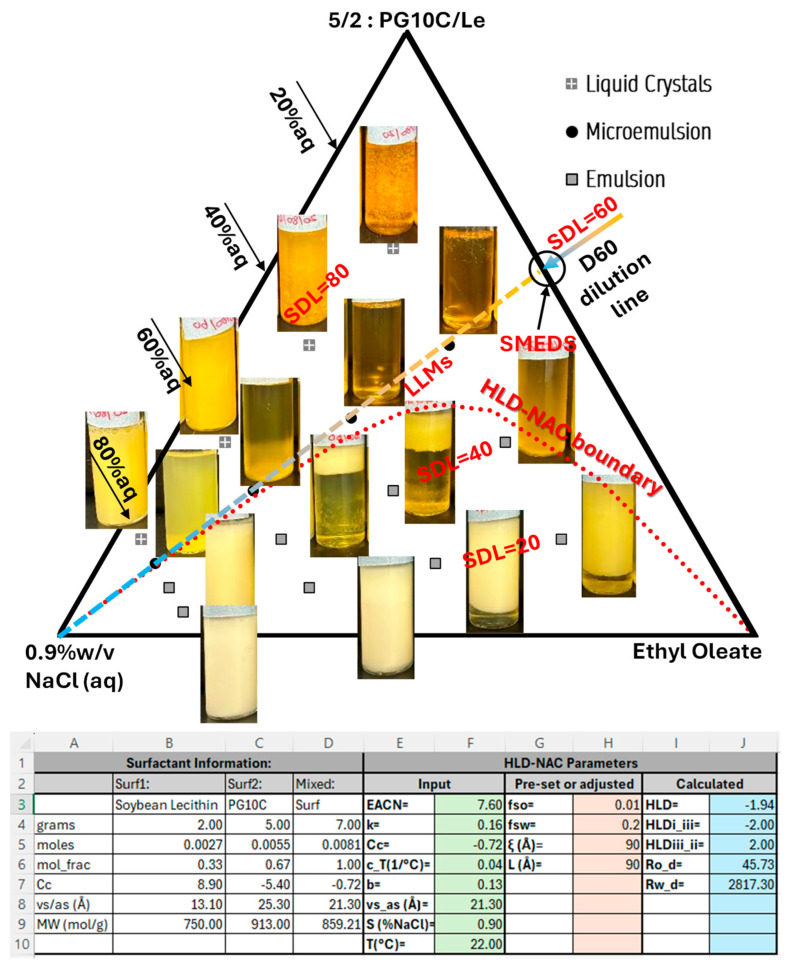
Ternary phase diagram (TPD) for PG10C + lecithin (5 to 2 mass ratio) as surfactant (top vertex), ethyl oleate as oil (right vertex) and an aqueous phase containing 0.9% NaCl (left vertex). SDL = 60 or D60 indicates 60% of total surfactant in mixture with ethyl oleate to produce the water-free SMEDS. Lecithin-linker µEs (LLMs) are SMEDS diluted with aqueous saline solutions. The screen shot at the bottom was from a modified version of the HLD–NAC tutorial Excel file used to predict HLD–NAC boundary, shown as a dotted line [16]. The algorithm for this calculation is shown in Figure 5.

The composition scan in Figure 3A pointed to 70 parts HL (PG10C) to 30 parts Le as a possible composition to explore as candidate SMEDS. The composition used to develop the TPD was slightly different, 5 parts HL to 2 parts Le (equivalent to 71.4% PG10C), where the slightly higher HL content was expected to contribute to preventing the formation of metastable phases. Figure 4 presents the TPD obtained experimentally, along with pictures of the vials obtained at the different compositions explored. The black dots in Figure 4, corresponding to the D60 dilution line (SDL = 60), show the fully dilutable single phase µEs (the LLMs). The pictures of the vials at SDL = 60 containing 60% and 80% aqueous solution appear as two-phase systems, but a minor swirl turns the entire system into a homogeneous and clear single phase, suggesting that these systems are close to the multiple-phase boundary but solubilize all the oil and water. The systems at SDL = 80% are highly viscous and show birefringence when set in cross-polarized light filters, suggesting the presence of hexagonal or lamellar liquid crystals. The white phases observed at SDL = 20 and 40 indicate the presence of stable emulsions of unsolubilized oil.

**Figure 5:**
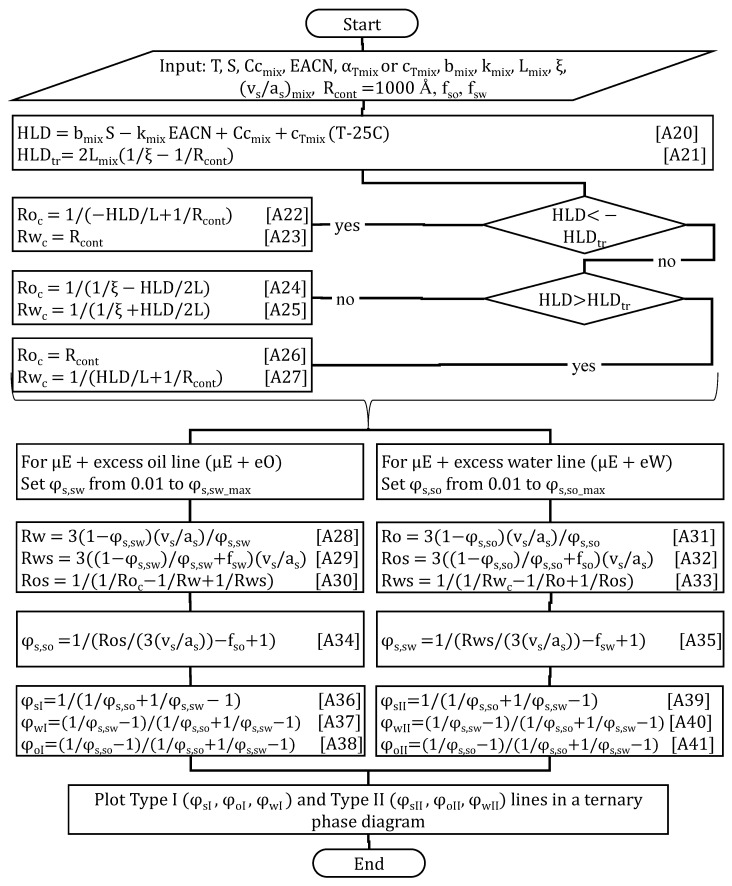
HLD–NAC algorithm to calculate the µE solubilization boundaries with excess oil (µE + eO) and with excess water (µE + eW) in a ternary phase diagram (TPD).

The dotted line in Figure 4 shows the HLD–NAC-predicted phase boundary for µEs coexisting with an excess oil phase (µE + eO lines) obtained using the input parameters shown in the screenshot included at the bottom of Figure 4. This screenshot was taken from a modified version of the HLD–NAC spreadsheet included in the HLD–NAC tutorial [16]. Figure 5 shows the algorithm used to generate the TPD lines. The input of the algorithm are the temperature (T), salinity of the system (S), oil EACN, and the mixed HLD–NAC parameters for the selected composition (x_2_): the characteristic curvature (Cc_mix_), volume to area ratio ((vs/as)_mix_), salinity factor (b_mix_), EACN factor (k_mix_), temperature factor (c_Tmix_), tail length (L_mix_), and the µE characteristic length (ξ) and fraction of the surfactant volume that contributes to the aqueous (f_sw_) and oil (f_so_) volume compartments. The algorithm requires the solution of the HLD–NAC in highly diluted conditions. This highly diluted case requires setting a high solubilization radius for the continuous phase (R_cont_ = 1000 Å, and this becomes Ro_cont_ or Rw_cont_, depending on the HLD).

Equation (A20) in Figure 5 is used to calculate the HLD of the system. Equation (A21) is used to calculate the transition HLD_III–II_ (HLDtr = HLD_III–II_) under dilute conditions, and under dilute conditions HLD_I–III_ = −HLDtr. Equations (A22) and (A23) calculate the solubilization radii under dilute conditions for oil (Ro_d_) and water (Rw_d_), respectively, in Type I µEs. Equations (A24) and (A25) are used to obtain Ro_d_ and Rw_d_ in Type III µEs. Equations (A26) and (A27) are used to obtain Ro_d_ and Rw_d_ in Type II µEs. After obtaining Ro_d_ and Rw_d_, there are two solubilization boundary lines that one can obtain; one is the saturated µE coexisting with excess oil (µE + eO) line, and the line of saturated µE coexisting with excess water (µE + eW). For the µE + eO line, one starts by setting the volume fraction of the surfactant mixture as if all the surfactant was only present in the aqueous phase (φ_s_sw_ = v_surf_/(v_surf_ + v_water_)) between 0.01 and a maximum value (φ_s_sw,max_). The value of φ_s_sw,max_ can be found by trial and error because, when φ_s_sw_ > φ_s_sw,max_, a portion of the predicted boundary line (beyond φ_s_sw,max_) produces unrealistic results. The published tutorial spreadsheet [16] contains a protocol to find φ_s_sw,max_. After setting φ_s_sw_, the solubilization radius of water (Rw) is found with Equation (A28). Equation (A29) calculates the radius of solubilization of water, including the surfactant contribution to the aqueous phase compartment (Rws). To construct TPDs, one uses the principle that the net curvature is the same under dilute (Hn = 1/Ro_d_ − 1/Rw_d_) or concentrated (Hn = 1/Ros − 1/Rws) conditions. Equation (A30) uses 1/Ro_d_ − 1/Rw_d_ = 1/Ros − 1/Rws to calculate Ros with the previously calculated Ro_d_, Rws, and Rw_d_ = Rw. With the value of Ros, one can now calculate the surfactant volume fraction in the oil phase, as if all of the surfactant were only present in the oil phase (φ_s_so_ = v_surf_/(v_surf_ + v_oil_)). Once one obtains φ_s_so_ for the set value of φ_s_sw_, one can find the volume fraction of the surfactant in the system (φ_sI_ = v_surf_/(v_surf_ + v_water_ + v_oil_)) using Equation (A32), the volume fraction of water in the system (φ_wI_ = v_water_/(v_surf_ + v_water_ + v_oil_)) using Equation (A33), and the volume fraction of oil in the system (φ_oI_ = v_oil_/(v_surf_ + v_water_ + v_oil_)) using Equation (A34). By setting a range of φ_s_sw_ values and repeating the steps in (A28) through (A34), then a set of compositions (φ_sI_, φ_oI_, φ_wI_) can be obtained and plotted to obtain the µE + eO line. This is what led to the red dotted line in Figure 4. A similar set of calculations can be performed for the µE +eW line (Equations (A35) through (A41)), but, since the system of Figure 4 is at the I-III boundary, only the µE +eO line is relevant.

For the system in Figure 4, the only adjusted parameters in the algorithm were the fraction of the surfactant volume that contributes to the volume of the “aqueous” (f_sw_) and “oil” (f_so_) compartments. To illustrate the meaning of f_sw_ and f_so_, let us consider a system containing 3 mL of oil, 2 mL of surfactant, and 5 mL of water. If f_sw_ = 0.4 and f_so_ = 0.1, the volume of the aqueous compartment is 5 mL (from water) + 2 × 0.4 mL (from the surfactant) = 5.8 mL, and the volume of the oil compartment is 3 mL (from oil) + 2 × 0.1 mL (from the surfactant) = 3.2 mL. Initially, it was thought that f_sw_ + f_so_= 1, but, after fitting various TPDs, it has become evident that this sum is often less than 1 [9,13]. For the HLD–NAC predicted boundary in Figure 4, f_so_ was set to 0.01 because any higher value would move the boundary beyond SDL = 60, and f_sw_ was set to 0.2 because any higher value would predict that, at SDL = 40 and 20% aqueous phase, the system would be a single phase µE. The physical significance of f_sw_ + f_so_ ≤ 1 needs further exploration, but it suggests that a portion of the volume of the surfactant cannot be used to accommodate the curvature of the oil and aqueous compartments.

### 2.4. Conductivity and Viscosity Along SDL = 60 (D60) Dilution Line

**Figure 6:**
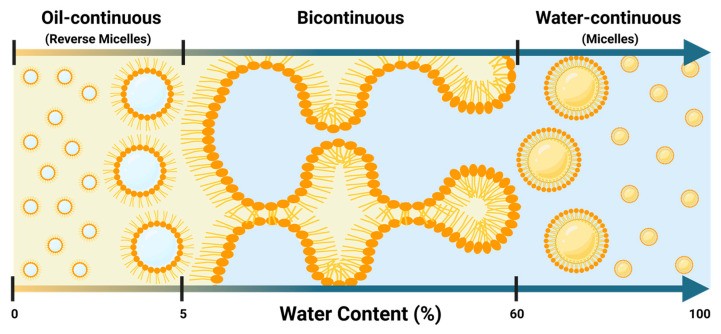
Schematic representation of lecithin-linker µEs (LLMs) transitioning from oil-continuous (reverse micellar) structures to bicontinuous phases at approximately 5% water content and from bicontinuous to micellar (water-continuous) systems at around 60% water content, according to the information in Figure 7 (Created in BioRender. Doratt, J., accessed on 4 January 2025, https://BioRender.com/a21a732).

According to Nouraei et al. [9,13], fully dilutable lecithin-linker microemulsions can form three distinct microenvironments as the aqueous phase increases—reverse micellar, bicontinuous, and micellar—whose transitions are reflected in conductivity and viscosity measurements. These properties help to experimentally identify when the system moves between oil-continuous, bicontinuous, and water-continuous phases. Figure 6 provides a visual representation of these microenvironments. One aspect that should be clarified is that, lecithin in SMEDS, due to the presence of HL and oil, does not exist in the form of rigid layers like those in liposomes or bilayers.

**Figure 7:**
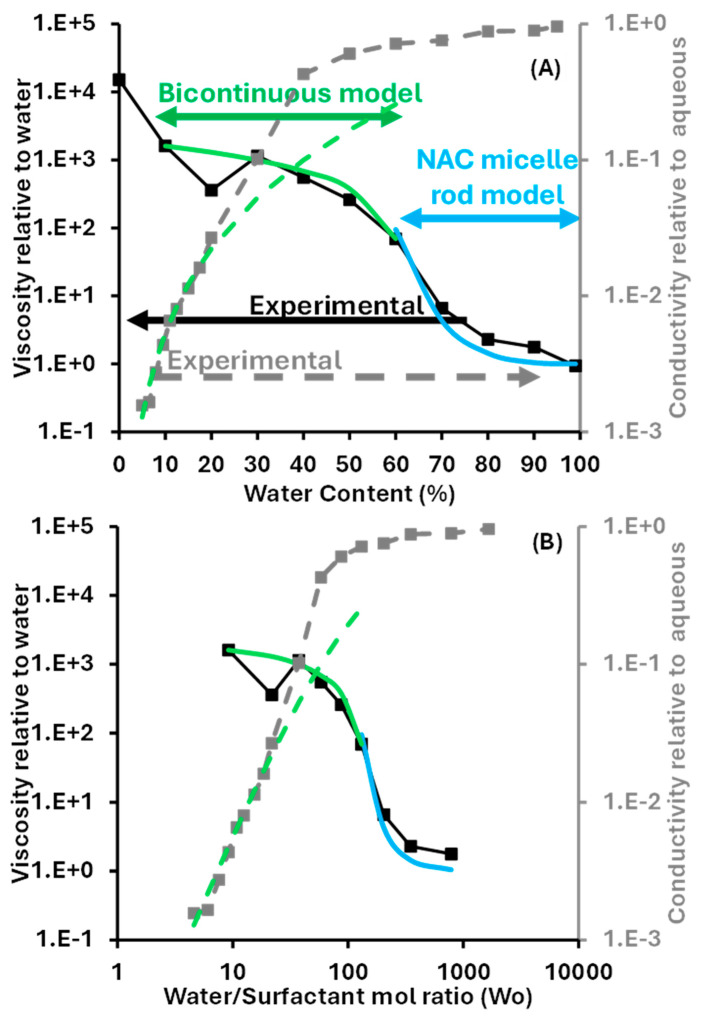
Relative conductivity (dashed line) and viscosity (solid line) of fully dilutable µEs (SDL = 60 or D60 in Figure 4) as a function of (**A**) water content expressed as volume % in the system, and (**B**) water to surfactant molar ratio (Wo). The green lines represent the conductivity and viscosity predicted bicontinuous polymer models, and the blue line represents the relative viscosity predicted via the HLD–NAC model using the dilute rigid rods model.

Figure 7 shows the relative conductivity and viscosity of LLMs (SDL = 60 in Figure 4) as a function of the aqueous phase content (Figure 7A) and the water-to-surfactant molar ratio (Wo, Figure 7B). The reference conductivity was the conductivity of water containing the same electrolyte concentration (based on total volume) as the diluted µEs, and the reference viscosity was the viscosity of water at 20 °C. The green lines were obtained using equations developed for bicontinuous polymer systems. The relative conductivity (σ_relative_) was predicted as [19]:

(4) $$\frac{\sigma_{\mathrm{bicontinuous}}}{\sigma_{\mathrm{aqueous}}}=\sigma_{\mathrm{relative}}=\frac{\varphi_{\mathrm{aqueous}}}{\tau }=\frac{\varphi_{\mathrm{aqueous}}}{\left(\frac{2-\varphi_{\mathrm{aqueous}}}{\varphi_{\mathrm{aqueous}}}\right)\;}$$

The term φ_aqueous_ is the volume fraction of the aqueous phase, and τ is the tortuosity. The tortuosity expression included in the last term of Equation (4) is the Mackie–Meares equation for ion transport in polymer networks [20]. The dashed green line shows the predicted relative conductivity using Equation (4).

According to Figure 7, for systems containing up to 20 wt% aqueous phase, the relative conductivity predicted by Equation (4) matches the experimental data. It is important to keep in mind that Equation (4) was obtained by combining two equations derived for bicontinuous polymers, where the polymer network is immobile. On the other hand, µEs are dynamic systems where the oil and water domains are constantly fluctuating. This suggests that, at least for systems with less than 20% aqueous phase, the mobility of the aqueous environment is rather limited, but, after 20% water, the larger experimental conductivity can be explained by the greater mobility of µE environments compared to fixed bicontinuous polymeric environments.

Considering that Equation (4) was designed for bicontinuous systems, the close match with the experimental data at low water content suggests that, even with as low as 5% aqueous content, the µEs are bicontinuous. The HLD–NAC can help evaluate if, conceptually, bicontinuous µEs are even possible at this low water content. To this end, we need to consider the solubilization radii for oil and water, including the surfactant volume contribution, Ros and Rws [9]:

(5) $$\mathrm{Ros}=3\left(\frac{\phi_{o}}{\phi_{s}}+f_{\mathrm{so}}\;\right)\left(\frac{v_{s}}{a_{s}}\;\right)\;;\;\mathrm{Rws}=3\left(\frac{\phi_{w}}{\phi_{s}}+f_{\mathrm{sw}}\right)\left(\frac{v_{s}}{a_{s}}\right)$$

When one obtains a single phase µE (i.e., a composition above the solubilization boundary line) the net curvature (Hn) is no longer controlled by HLD but is calculated with Equation (2) using the solubilization radii of Equation (5); Hn = (1/Ros − 1/Rws). Similarly, the average curvature is calculated using Equation (3) with the solubilization radii of Equation (5); Ha = (1/2)·(1/Ros + 1/Rws). With Hn and Ha, the size and shape of oil-swollen micelles (positive Hn) or water-swollen reverse micelles (negative Hn) can be calculated, assuming that these swollen micelles or reverse micelles have a cylindrical body with hemispherical caps. The radius (R_d_) and length (L_d_) of the cylinders is calculated as [21]:

(6) $$R_{d}=\frac{2\mathrm{Ha}-\sqrt{4{\mathrm{Ha}}^{2}-2\mathrm{Ha}\cdot \left|\mathrm{Hn}\right|}}{\mathrm{Ha}\cdot \left|\mathrm{Hn}\right|}$$

(7) $$L_{d}=2\cdot R_{d}\cdot \left(2-\frac{\left|\mathrm{Hn}\cdot R_{d}\right|}{\left|\mathrm{Hn}\cdot R_{d}\right|-1}\right)$$

For a system with near zero curvature, when |Hn| < 1/R_d_, Equation (7) produces negative L_d_ values, signifying that the system cannot exist as a water-continuous or oil-continuous system, and instead, a bicontinuous µE is obtained. For the D60 (SDL = 60) dilution line, with a given level of water dilution, for example, 20% water (φ_w_ = 0.2), the surfactant fraction is φ_s_ = (1 − φ_w_)·SDL/100 = 0.8 × 0.6 = 0.48; then, for the oil, φ_s_ = (1 − φ_w_)·(100 − SDL)/100 = 0.32. Using f_so_ = 0.01, f_sw_ = 0.2, and v_s_/a_s_ = 21.3 Å (see Figure 4 bottom—NAC input table), and these volume fractions, Ros and Rws are calculated using Equation (5), then Hn and Ha are calculated. Using Equations (6) and (7), R_d_ and L_d_ are calculated. Using this procedure, negative L_d_ values were obtained along the D60 dilution line, from 5 wt% water to just below 60% water, suggesting that all of these systems are bicontinuous. This NAC prediction is consistent with the close match between the experimental relative conductivity and the relative conductivity predicted by the bicontinuous polymer model (Equation (4)).

The radius (R_d_) and length (L_d_) of the swollen micelles and reverse micelles along the dilution line can also be used to predict the viscosity of the µEs, assuming that they behave as dilute rods [21]:

(8) $${µ}_{\mathrm{relative}}=\frac{{µ}_{µE}}{{µ}_{0}}=\left(1+\frac{\phi_{\mathrm{solubilized}}}{\pi }{\left(\frac{L_{d}}{R_{d}}\right)}^{2}\right)$$

The term µ_µE_ is the µE viscosity, µ_0_ is the viscosity of the solvent (water for oil-in-water µEs), and φ_solubilized_ is the volume fraction of the solubilized phase (the oil, and the surfactant contribution to the oil compartment, for oil-in-water µEs). The solid blue line in Figure 7 was obtained using Equation (8). The close match between the predicted relative viscosity (no fitting parameters) and the experimental relative viscosity for a water content of 60% or more suggests that the NAC model accurately captures the changes in the size and shape of oil-swollen micelles along the dilution line. This observation is consistent with previous evaluations that considered not only relative viscosity but also particle size evaluated using dynamic light scattering and neutron scattering [9,13,21]. To predict the relative viscosity in the bicontinuous region (solid green lines in Figure 7), a linear bicontinuous polymer melt model was used [22]:

(9) $${µ}_{\mathrm{relative}}=\frac{1}{{µ}_{\mathrm{reference}}}\;\left({µ}_{1}\phi_{1}+{µ}_{2}\phi_{2}\right)$$

The reference fluid for the relative viscosity in Figure 7 is water at 20 °C (µ_ref_ = 1 mPa·s). Fluid 1 was taken as the µE obtained with 10% water (µ_1_ = 1600 mPa·s), and fluid 2 was taken as the µE obtained with 60% water (µ_2_ = 70 mPa·s). Overall, this linear approximation matches well with the experimental values, except for the dip in measured viscosity at 20% water. A second set of viscosity measurements was undertaken with systems containing 8% yeast, and the viscosity obtained was nearly identical to those obtained from yeast-free systems, except for the systems with 70% water or more, where the viscosity of the yeast suspension was two to three times larger than the viscosity of the yeast-free system. The dip in viscosity at 20% water was also observed in this second set of measurements. At this point, the origin of the dip in viscosity at 20% water is not clear and should be explored in future studies.

### 2.5. Yeast Dispersibility

**Figure 8:**
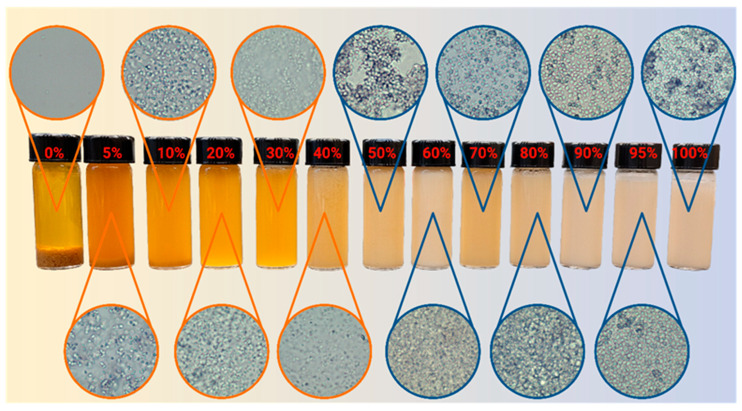
Dispersibility of 8% yeast suspension in LLMs produced along the D60 (SDL = 60) dilution line in Figure 4. The percentage indicated corresponds to the percentage of the aqueous phase in the LLM. The circles show the bright field micrographs obtained using a 20× objective.

Figure 8 illustrates the dispersibility of yeast (*Saccharomyces cerevisiae*) in LLMs obtained along the D60 dilution line, with an increasing aqueous phase from 0% to 100%. At a 0% aqueous phase, the yeast was unable to disperse, settling at the bottom of the vial. When a sample was taken from the middle of the vial, 15 min after 8% of yeast was dispersed, no yeast cells were observed, suggesting that yeast may not be activated in water-free systems and remain dormant in a dry state. At a 5 wt% aqueous phase, a significant portion of the cells remained suspended in the system, suggesting that yeast was activated in those systems. Yeast cells require a water activity of at least 0.61 to be activated [23]. Although water activity was not measured, previous studies with ionic µEs reveal that, at water-to-surfactant mol ratio (Wo) as small as 2.5 (~3 wt% aqueous in LLMs), the activity of water is 0.79 [24]. This suggests that, for LLMs with 5% water, yeast cells are activated (and suspended in the process), which is to be expected considering that instant dry yeast cells were used in this study. Full and stable yeast dispersion was achieved from 20% aqueous content and above. From 30 to 60 wt% aqueous, the micrographs showed yeast clusters in bicontinuous µEs. For the water-continuous µEs (>60 wt% aqueous), the cells were homogeneously dispersed.

### 2.6. Yeast Viability and Activity

**Figure 9:**
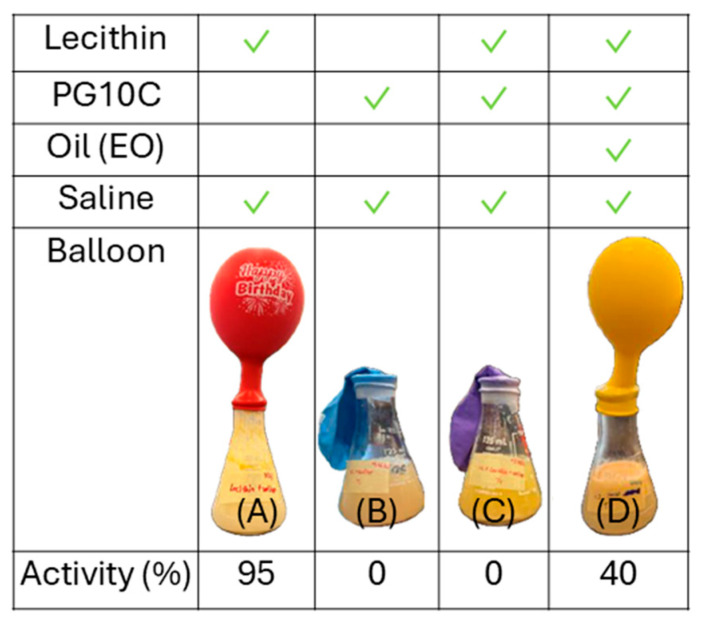
Relative yeast activity in control samples: (**A**) 3.4 parts Le/80 parts (by mass) saline, (**B**) 8.6 HL (PG10C)/80 saline, (**C**) (3.4 Le + 8.6 HL)/80 saline, (**D**) (3.4 Le +8.6 HL+ 8 parts oil (ethyl oleate)/80 saline. The composition of (**D**) corresponds to an 80% aqueous dilution along the D60 line in Figure 4. Each of these systems containing 8% yeast were left for 24 h before mixing with a sucrose solution (25 g suspension + 75 g sucrose). The balloons show the CO_2_ production after another 24 h. The relative activity compares the volume of CO_2_ produced in each case and the volume produced by a suspension of 8% yeast in saline solution (0.9% *w*/*v* NaCl).

Figure 9 shows the relative activity of 8% yeast suspensions in aqueous solutions of lecithin (Le, system “A”), hydrophilic linker PG10C (HL, system “B”), Le + HL (system “C”), and in an LLM (Le + HL + EOL, system “D”) produced along the D60 dilution line with the 80% aqueous phase. This 80% aqueous phase dilution was selected as a reference point because it produced well-dispersed cell suspensions in aqueous-continuous environments. The relative activity was calculated in reference to the CO_2_ production by an 8% yeast suspension in a saline (0.9% *w*/*v* NaCl) solution. The data in Figure 9 show that lecithin does not significantly reduce the activity of yeast. It should be clarified that lecithin on its own can only be dispersed in the saline solution and is not fully dissolved. The system with hydrophilic linker (HL, PG10C) and the system with the mixture of HL and Le deactivated the yeast. Microscope images did not show signs of mechanical damage to the cells; however, it is known that surfactants can fluidize cell membranes, an effect that is similar to heat-induced cell death [25,26].

Hydrophilic linkers have been reported to reduce interfacial rigidity substantially [10]. This may explain the strong deactivating effect of HL alone or in combination with lecithin. However, once the full µE (LLM) is produced with the addition of EOL (the oil), then a substantial fraction of the activity is still retained. This partial activity may be explained by the fact that the presence of the oil creates an oil-water interface where the HL can be adsorbed, discouraging HL from primarily adsorbing on the surface of cell membranes. In other words, the addition of EOL likely reduced the activity of the HL in the aqueous phase.

**Figure 10:**
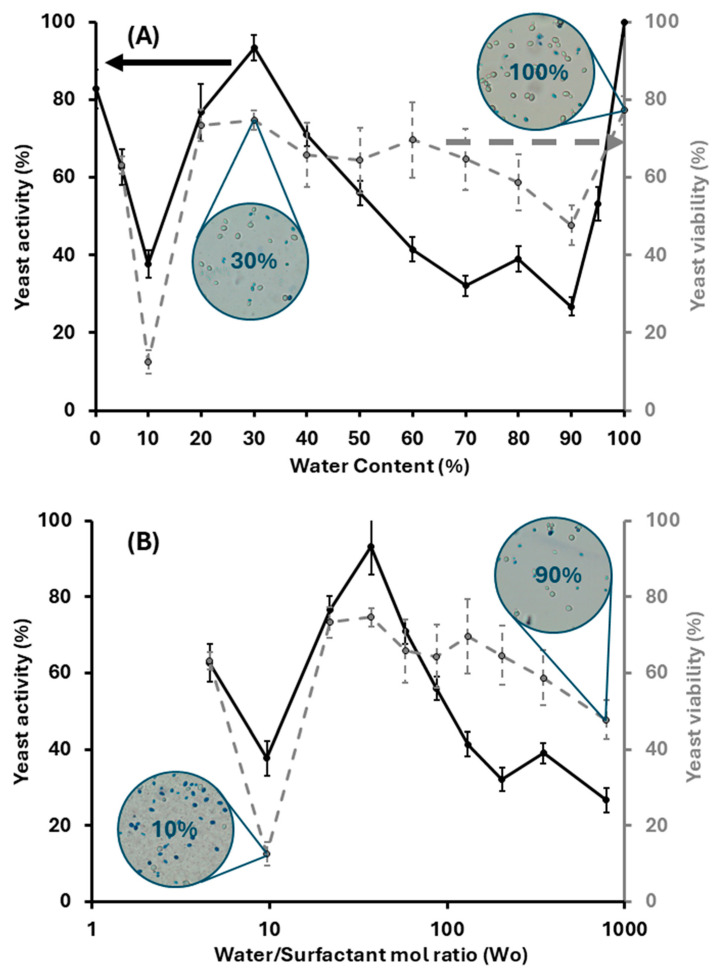
Yeast activity and viability as a function of water content (**A**) and water-to-surfactant ratio, Wo, (**B**)—along the dilution line D60 illustrated in Figure 4. The left vertical axis (black solid line) represents yeast activity, measured by the amount of CO_2_ produced over 24 h after feeding the yeast-loaded (loading: 8% *w*/*w*) LLMs (previously exposed to the formulation for 24 h with a 25% *w*/*v* sucrose solution. The right vertical axis (gray dashed line) shows yeast viability following the same exposure and feeding protocol. The viability was determined by staining the dead yeast cells with a 0.1% aqueous methylene blue, as shown in the micrographs for the LLM systems containing 10%, 30%, 90% and 100% of the aqueous phase.

Figure 10 presents the activity and viability (assessed via the methylene blue staining method) of yeast suspensions in LLMs along the D60 line. The aqueous phase content is presented in Figure 10A as wt% and in Figure 10B as water/surfactant mole ratio (Wo). At 0% aqueous content (i.e., the SMEDS at D60), the yeast exhibited relatively high activity, producing the equivalent of 82.7 ± 5.0% of the CO_2_ produced by yeast suspended in saline solution (the reference suspension) for 24 h before the addition of the sucrose solution. Considering that yeast was not dispersible in SMEDS, suggesting that yeast was not activated, the large activity obtained once the sucrose solution was added indicates that SMEDS served as a dry preservation media for yeast. In LLMs with 5% aqueous content, the viability was 59.9 ± 2.8%, and the activity was 62.6 ± 4.5%, suggesting that a significant portion of the yeast cells remained alive despite partial dispersion observed in Figure 8 for this system. As the aqueous content increased to 10% (Wo~10), the activity and viability drastically decreased to 37.7 ± 3.5% and 23.8 ± 1.7%, respectively, but the activity and viability bounced back to 76.7 ± 7.4% and 71.2 ± 4.0%, once the aqueous content reached 20% (Wo~22). Yeast activity reached a peak (93.3 ± 3.2%) in LLMs with 30% aqueous content (Wo~37) while maintaining a relatively high viability (75.1 ± 2.1%). From there, further increases in water content in the LLM resulted in a gradual decrease in activity and viability, reaching a plateau of about 30% activity once the LLM reaches a 70% aqueous content. The viability also reduces slightly, but, in most cases, the viability remains above 50%.

Hopper et al. investigated the photosynthetic activity (measured as O_2_ generation) of cyanobacteria and green algae as a function of Wo in Tween 85-Span 80-hexadecane µEs, finding that there was no activity when Wo was less than 50 and maximum activity at Wo~80 to 1802. The authors posed that Wo > 50 was needed to activate the cells; however, for yeast, even Wo~3 should be enough to activate the cells. The high activity found at Wo~0 suggests that, in a dry state, the yeast remains dormant in SMEDS but is quickly activated once in contact with the sucrose solution. The minimum activity and viability found at Wo~10 in Figure 10B suggests that, during the 24 h incubation of yeast in the LLM with 10% aqueous, there was some level of metabolic activity (supported by the evidence of metabolic activity in the sugar-free systems in Figure 3B) that produced metabolites (possibly alcohols and fatty acids), but, given the small amount of water and the poor transport, these metabolites could have concentrated to toxic levels [27].

As the aqueous content in the LLM increases beyond Wo~10, the increased volume of water solubilized likely helped dilute and transport metabolites (illustrated by the increased conductivity in Figure 7B when Wo > 10) during the 24 h incubation period before the yeast was set in contact with the sucrose solution.

Much like Hopper et al., we were not able to identify a clear reason for the existence of an optimal Wo for cell activity (Wo~37 in our case). At Wo less than 37, increasing Wo increases the transport of aqueous metabolites (Figure 7B), but, after Wo~37, there are only marginal increases in conductivity and transport through the aqueous phase. On the other hand, increasing Wo decreases the amount of SMEDS (Le + PG10C + EOL) in the LLM, which means it decreases the ratio of SMEDS to yeast. The data suggest that the Le–HL–EOL system plays a role in kickstarting the metabolic processes (Figure 3B) in yeast. This observation is consistent with a previous study where a Le-PG10C-Span80 formulation designed as an oil spill dispersion media maintained the biodegradation in a nutrient-deprived cell growth media, suggesting that the carbon, phosphor, and nitrogen in lecithin served as nutrients for the cells [15]. Another study showed that the addition of small amounts of lecithin could improve alcohol production in yeast fermentation, which was explained in terms of lecithin being used as a lipid source to form new cell membranes (aid in yeast cell division) [28]. The idea that the SMEDS lipids help kickstart the fermentation in yeast is also supported by the findings of Pfammatter et al., who observed a 4-fold increase in cell counts in the first 24 h that yeast cells were exposed to Tween85-Span80-hexadecane µEs containing 3% of an aqueous 0.9% *w*/*v* NaCl solution without added nutrients [3]. In fact, their work shows that, when the yeast (*C. pseudotropicalis* in their case) was exposed to 0.9% *w*/*v* NaCl solution without surfactants and without nutrients, there was only about 1.5-fold increase in cell counts after 24 h of incubation. The same work showed, using C^14^-labeled hexadecane, that, during the incubation period, some of this oil was used as a carbon source for aerobic respiration.

Another factor to consider when interpreting Figure 10 is that hydrophilic linkers, like PG10C, tend to remain partially present in the aqueous phase even in LLMs10. Therefore, the more water in the system (increasing Wo), the more hydrophilic linker (PG10C) will end in the aqueous phase. As we learned from Figure 9, when PG10C is in the aqueous phase, PG10C tends to suppress yeast activity. This also explains why the LLM with 90% aqueous phase has the lowest yeast activity, but with 100% aqueous (only saline solution, no Le-PG10C-ethyl oleate), then, the activity is the largest observed with these systems.

### 2.7. Long-Term Storage in SMEDS and LLMs

**Figure 11:**
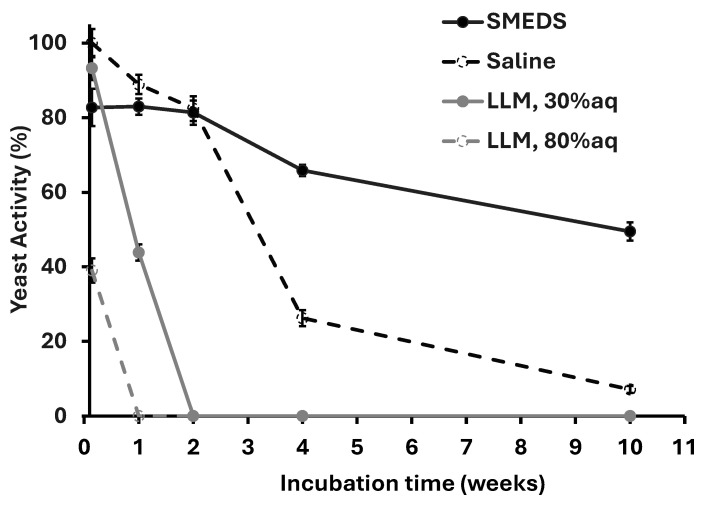
Yeast activity in LLMs (measured by the amount of CO_2_ produced after exposure to sucrose solution) versus the incubation time of the yeast in SMEDS or LLMs before contacting with the 25% sucrose solution.

The activity of yeast in SMEDS and selected LLMs was evaluated as a function of incubation time (the time before the yeast + LLM or SMEDS was exposed to the sucrose solution) to determine how long one could store yeast in these systems before there is a significant decrease in activity, assessed in terms of CO_2_ production. Figure 11 presents the activity of yeast in LLMs containing 0% (SMEDS), 30%, 80%, and 100% (only aqueous phase) 0.9% *w*/*v* NaCl solution.

The system with 100% aqueous phase produced the largest activity after one day of incubation. However, as the incubation time increased over 2 weeks, the activity reduced substantially. This decline may indicate that, despite the stabilizing effect of the saline environment, the absence of nutrients over time gradually impaired cellular functions and viability, resulting in diminished metabolic activity. The LLMs with 80% and 30% aqueous content show a precipitous decrease in activity that is unlikely to be associated with the lack of nutrients as Le and EOL serve that function, but rather the problem of metabolite build-up and the presence of the hydrophilic linker (PG10C) in the water that tends to suppress yeast activity, as shown in Figure 9.

For the LLM with 30% aqueous phase, it is remarkable that the yeast was still active after 1 week of incubation, answering part of the research question that LLMs can extend the survival of unacclimatized yeast beyond the 3 days reported for other µE formulations1. The D60 SMEDS (0% LLM) shows a gradual decline in activity, decreasing from 82.73 ± 4.99 in Week 0 to 49.50 ± 2.46 by Week 10. This observation then answers the second part of the research question that SMEDS can substantially extend the survival of unacclimatized yeast beyond the 3 days reported for previous µE formulations. The slow decrease in yeast activity in SMEDS, compared to the LLMs, is likely associated with the low water activity in the D60 SMEDS since yeast cells are known to remain dormant when the water activity is lower than 0.61 [23]. Recent work shows that when yeast cells enter a dormancy stage due to dehydration (the process used to make dry powder yeast), the cells enter a solid-like state triggered by an acidification of the cytoplasm that results in a widespread macromolecular assembly of proteins and sugars, causing the cytoplasm to gel [29]. Since dry powder yeast cells are added to SMEDS (no water added), then, as long as the SMEDS remain dry, the lack of water keeps the yeast dormant. The gradual loss of yeast activity in SMEDS might be associated with water absorption from air over the 10-week period since the formulation was stored in ambient air (T~25 °C, ~50% relative humidity), protected with Parafilm™ film, which has low but non-zero water vapor permeability [30]. One fundamental difference between dry yeast powder (that remains stable for one year or more if the package seal is not compromised) and the yeast in SMEDS is that the yeast powder does not have nutrients, thus absorption of water from air is not likely to result in activation. However, in SMEDS, lecithin and EOL are nutrients, and the absorption of water from air is likely to result in premature yeast activation. Future stability tests are planned to keep track of water activity in SMEDS under different storage conditions and its impact on cell viability and activity. If water absorption is involved, then appropriate storage and packing/sealing must be considered.

Another potential explanation for the gradual decrease in yeast activity in SMEDS is the potential effect of the hydrophilic linker (HL) on the yeast cell wall. The prolonged exposure of dormant cells to the HL in SMEDS could allow enough time for HL molecules to diffuse into cell membranes, fluidizing the membrane, following a similar deactivation mechanism proposed to explain the results in Figure 9B,C [25,26]. The viability of dry dormant cells diminishes when the membranes are fluidized and is often reflected in lower glass transition temperature of the cell membrane [30,31]. Future stability tests are planned to keep track of the effect of HL on cell membrane fluidity, assessed via glass transition temperature, for cells stored in SMEDS. If HL-induced membrane fluidization is involved, then alternative formulations with different HL, or different HL/Le ratios, or membrane protectants, such as albumin, could be considered, as well as producing acclimatized cells that are resistant to the potential HL effects.

One additional factor that was not fully considered in this work was the effect of shear on cell death induced by mechanical stresses. The mixing used in this work was relatively mild (vortex-mixing), and no cell rupture was observed in any of the images, even in systems with low cell viability. However, the use of high-shear processing technologies, such as high-pressure homogenization, can lead to mechanically induced yeast cell rupture [32]. For such cases, the lubrication phenomena in localized high shear environments are likely to play a role, and the potential effects of surfactants, and µEs, on lubrication should be considered. In some cases, surfactants have been shown to display “superlubricity effects” that might help yeast cells survive high shear environments [33].

## 3. Materials and Methods

### 3.1. Materials

The chemicals used in the formulation of the lecithin-linker microemulsions included MiliQ water (18 Ω) obtained from Milli-Q^®^ IQ 7000 Ultrapure Water Purification System, Sigma-Aldrich (Oakville, ON, Canada); ethyl oleate (98%), which was purchased from Fisher Scientific (Mississauga, ON, Canada); L_α_-granular soybean lecithin, granular (from soybean oil), which was purchased from Thermo Fisher Scientific-Across Organics (Mississauga, ON, Canada); sodium chloride (>99.0%), sucrose (≥99.5%), methylene blue hydrate (≥95.0%), and limonene (97%, racemic mixture), which were purchased from Sigma-Aldrich (Oakville, ON, Canada); polyglyceryl-10-caprylate (Polyaldo^®^ 10-1-CC, 97%), which was donated by Azelis Canada (Brampton, ON, Canada); surfactant pentaethylene glycol nonyl ether (C9E5 or Dehydol^®^ OD-5, 100% active), which was donated by BASF North America (Wyandotte, MI, USA); and *Saccharomyces cerevisiae*, which was purchased from a local store as baker’s yeast, Fleischmann’s^®^ quick-RISE instant dried yeast manufactured by AB Mauri Food Inc. (St. Louis, MO, USA). Plastic balloons were sourced from Pioneer Ballon Canada Ltd. (Hamilton, ON, Canada). All chemicals were used as received without further purification.

### 3.2. Methods

#### 3.2.1. Estimation of HLD and NAC Parameters for Lecithin-Linker Microemulsions

A salinity scan was conducted to estimate the EACN of ethyl oleate (EOL) using the surfactant C9E5 at 40 wt% in the aqueous phase, a water-to-oil ratio (WOR) of 1:1, and a total volume of 6 mL. For C9E5 concentrations of 20 wt% or higher, the characteristic curvature (Cc) was −0.3, determined via salinity scans with limonene as the oil phase. All vials were gently agitated and allowed to equilibrate for two weeks before any observations were recorded. Phase volumes were then analyzed using a modified HLD–NAC model Excel spreadsheet to fit EACN and ξ [16].

In a separate composition scan, the Cc of lecithin (Le) and the hydrophilic linker polyglyceryl-10-caprylate (PG10C) was assessed by mixing each surfactant with C9E5 (reference) and limonene (EACN = 6), at WOR = 1:1, total volume of 6 mL, and 20 wt% total surfactant. The aqueous phase salinity was 0.9% *w*/*v* NaCl. After two weeks of stabilization, phase volumes were again fitted with a modified HLD–NAC model to determine the Cc and ξ values.

#### 3.2.2. Lecithin–Hydrophilic Linker Phase Scans

To determine the HLD–NAC parameters (L and ξ in Equations (2) and (3)) for the Le + HL (PG10C) system with ethyl oleate, a composition scan was carried out at 20 wt% total surfactants in the aqueous phase, varying the HL from 10% to 90% in 10% increments. The water-to-oil ratio (WOR) was kept at 1:1 (6 mL total), with a salinity of 0.9 g NaCl per 100 mL in the aqueous phase (S = 0.9%). After two weeks of room-temperature equilibration, most samples still formed gel-like emulsions. They were then heated to 80 °C for one hour (no mixing), cooled, and allowed to equilibrate for another week; this cycle was repeated four times before images were taken. The phase volumes were fitted with a modified HLD–NAC model.

Since the yeast activity involves the use of sucrose as a substrate, it is important to assess whether sucrose affects the Le + HL microemulsion phase behavior. The effect of sucrose on the Le + HL microemulsion phase behavior was determined by repeating the scan described above with the addition of 25% *w*/*v* sucrose in the aqueous phase (still at S = 0.9%). After taking pictures and observations of the scans containing the sucrose, 0.15 g of baker’s yeast (5 wt% in the aqueous phase) was added to each vial to gauge whether yeast metabolism influences the phase behavior of the Le + HL microemulsions.

#### 3.2.3. Construction of the Ternary Phase Diagram (TPD)

To construct the ternary phase diagram and determine the minimum surfactant concentration for a fully dilutable pathway, binary mixtures were prepared along the surfactant–oil axis of the TPD by varying the total surfactant (Le + HL) to ethyl oleate (EOL) weight ratio from 10% to 90% respectively (or vice versa). These surfactant–oil blends, without added water, serve as self-microemulsifying delivery systems (SMEDS) candidates. The percentage of surfactant in the surfactant–oil mixture is referred to as the Surfactant Dilution Line (SDL); for instance, SDL = 60 corresponds to 60% surfactant and 40% oil (the D60 line for simplicity). The surfactant blend (lecithin + polyglyceryl-10 caprylate) was held at a 2:5 ratio, identified from prior phase scans.

To create dilution lines between 5% and 95% (*w*/*w*) aqueous phase, a 0.9% *w*/*v* NaCl solution was added to each surfactant–oil mixture in glass vials, followed by gentle hand shaking. The dilution behavior was observed within two hours and after two weeks of equilibration. Samples that remained as clear, single-phase solutions without birefringence under cross-polarized light were classified as microemulsions (μEs). These results were then mapped onto a pseudo-ternary phase diagram, with EOL at the right vertex, the surfactant blend at the top vertex, and the 0.9% *w*/*v* NaCl solution at the left vertex.

#### 3.2.4. Preparation of Le–HL µEs (LLMs) and Yeast-Loaded LLMs Along SDL = 60

Ethyl oleate, lecithin, and PG10C were mixed at 40%, 17.1%, and 42.9% *w*/*w*, respectively, using a magnetic stirrer at 80 °C for 30 min to ensure complete solubilization of lecithin and PG10C in ethyl oleate and form the D60 SMEDS. The D60 SMEDS was then diluted with the 0.9% *w*/*v* NaCl solution to produce a final aqueous content from 0% to 100% by weight, producing the LLMs. The solutions were mixed until clear, homogeneous mixtures were obtained. Each μE was labeled according to its aqueous content (e.g., 0%, 5%, 10%, etc.). Dry yeast (*Saccharomyces cerevisiae*) was rehydrated in the corresponding saline solution fraction of the sample for 15 min at room temperature prior to use (no rehydration was used for 0% aqueous). The rehydrated yeast slurries were then added to each μE to a final dried yeast concentration of 8.0% (*w*/*w*). The yeast-loaded microemulsion mixtures were thoroughly mixed using a vortex mixer for 2 min to ensure uniform distribution. Each sample was allowed to stand for at least 24 h at room temperature before any measurement was taken.

#### 3.2.5. Electrical Conductivity Studies

Conductivity measurements of single-phase μEs for SDL = 60 (D60) were obtained through a LabQuest 2 standalone interface (Vernier Science Education, Beaverton, OR, USA) equipped with a conductivity probe (CON-BTA, Vernier Science Education, USA). A control sample made of MiliQ water and increasing concentrations (0–100%) of saline solution (NaCl 0.9% *w*/*v*) was also measured. All dilution samples were measured in triplicates.

#### 3.2.6. Viscosity

The viscosity of the single-phase lecithin-linker μEs (D60 LLMs) and yeast-loaded μEs (D60 LLM/Yeast), diluted with an aqueous phase containing 0.9% *w*/*v* NaCl, was measured along the D60 path using a Carri-Med CSL2 rheometer (TA Instruments, New Castle, DE, USA). A 2-degree cone geometry with a diameter of 4 cm and a gap of 60 μm was employed. Temperature control was maintained at 25 °C using a Peltier plate. The measurements were performed in flow mode, with shear rates varying from 0.1 to 100 s^−1^.

#### 3.2.7. *Saccharomyces cerevisiae* Dispersibility in Le-HL μEs

The dispersibility of the yeast within each microemulsion formulation was assessed visually. After the 24 h incubation period, the samples were inspected for signs of yeast settling or sedimentation. Samples were classified into three categories: “Fully Dispersed”, “Partially Dispersed”, or “Not Dispersed”, based on the visual homogeneity of the mixture. Microscopy was conducted to evaluate the dispersibility of yeast within the LLMs. Small aliquots from each sample were placed onto glass slides (VWR Inc., Mississauga, ON, Canada) and covered with micro cover glass (VWR Inc., Mississauga, ON, Canada). Images were captured using an Olympus BX51 optical microscope equipped with an Olympus U-CMAD3 camera adapter (Olympus Corporation, Tokyo, Japan) and an AmScope MD35 camera (AmScope Company, Irvine, CA, USA) at 20× magnification. Microscopic images were analyzed to assess the dispersion of yeast cells, the presence of clusters, and any structural patterns within the µEs. Images were categorized based on the uniformity of yeast distribution and the presence of any clustering behavior.

#### 3.2.8. *Saccharomyces cerevisiae* Activity

The yeast activity test was adapted from Agee and Rowland [34] who developed the test as a demonstration lab for the activity of S. cerevisiae by keeping track of the volume of CO_2_ produced using balloons that inflate with the CO_2_ produced according to the well-known biochemical pathway for alcoholic fermentation involving yeast as a catalyst, which is expressed in Equation (10), where 1 mole of glucose turns into 2 moles of ethanol and 2 moles of carbon dioxide.

(10) $$C_{6}H_{12}O_{6}\overset{\;\mathrm{yeast}\;}{\to }\;2{\;C}_{2}H_{5}\mathrm{OH}\;+2{\;\mathrm{CO}}_{2}$$

Twenty-five grams of the LLM (including the SMEDS only, i.e., LLM with 0% aqueous) were added to 250 mL conical flasks. Dry yeast cells (2.0 g) were mixed with the LLMs. The following day, 75.0 g of a sucrose solution (25% *w*/*v* sucrose in 0.9% *w*/*v* NaCl) were added to the mixture. A helium-grade balloon was placed over the opening of the conical flask and sealed with Parafilm. The flask was gently shaken to ensure that the yeast was evenly distributed with no remaining lumps. After 24 h, the circumference of the balloon was measured and recorded to calculate the volume of CO_2_ produced during fermentation. The CO_2_ produced was taken as a measurement of cell metabolic activity.

#### 3.2.9. *Saccharomyces cerevisiae* Viability

The methylene blue test consists of exposing 50 µL of the LLM yeast suspension with 50 µL of a solution of 0.1 wt% methylene blue in water and incubating this mixture for 5 min [35,36]. Live yeast cells contain reductases that turn methylene blue colorless, which is something that dead cells cannot accomplish. After the incubation time, samples of the resulting suspension were observed using the Olympus BX51 optical microscope in brightfield mode with a 20× objective. The live and dead cells were manually counted, making sure that the total number of cells per image was at least 30. The percentage of live cells was calculated as the number of live cells (unstained) divided by the total number of cells times 100%. For each yeast-LLM suspension, at least five pictures per sample were taken, and two samples were evaluated. The average percentage of live cells and the standard deviation (shown as error bars) were reported.

## 4. Conclusions

This study explored the viability and activity of dry instant yeast (*Saccharomyces cerevisiae*) dispersed in fully dilutable lecithin-linker microemulsions (LLMs) with water content ranging from 0% (a water-free system known as self-microemulsifying delivery system or SMEDS) to LLMs containing 95% aqueous phase, and in 100% saline (0.9% *w*/*v* NaCl). The SMEDS contained a surfactant mixture of lecithin (Le) and polyglycerol-10-caprylate (PG10C) as hydrophilic linker (HL) at a ratio Le/HL of 2/5, which was obtained after conducting µE phase scans using ethyl oleate (EOL) as the oil phase. The weight ratio of the surfactant mixture to EOL in the SMEDS was fixed at 60/40 (also known as a D60 dilution line). This D60 dilution line was predicted using the HLD–NAC model with parameters derived from µE phase scans fitted using a modified version of a published HLD–NAC spreadsheet. The HLD–NAC model, combined with measurements of viscosity and electrical conductivity, showed that LLMs containing between 5% and 60% aqueous phase were bicontinuous in nature and that, for systems with less than 30% aqueous phase, the transport of ions and likely other water-soluble species was very limited.

LLMs with 10% aqueous phase produced the lowest yeast viability and activity, but LLMs with 30% aqueous phase produced the highest viability and activity. For the 10%, this minimum was interpreted as being caused by the yeast being partially active, generating metabolites, but that these metabolites concentrate in a water-confined environment, likely leading to metabolite-induced toxicity. At 30% aqueous, the LLM provides the nutrient benefits of the SMEDS, enough water to enable metabolite transport, but not too much water that would induce the release of PG10C (HL) into the aqueous phase. The studies showed that the presence of this HL in water had a detrimental effect on yeast activity, explaining the second minimum in yeast activity when the aqueous phase content reached 90%. When yeast was loaded in SMEDS, the cells were stable for a 10-week period that has not been reported before for yeast in microemulsions or regular emulsions. Having near-zero water activity allows the SMEDS to keep the cells dormant for an extended period. However, the gradual absorption of water, or the gradual diffusion of HL into the dormant cell membranes are possible causes for the gradual decrease in yeast activity in SMEDS.

The longer viability and activity observed in LLMs (>1 week) and SMEDS (~10 weeks), compared to previous studies (3 days) suggests that this approach could reopen the doors to using microemulsions as solvent media in various biotransformation processes.

## Data Availability

The data presented in this study are available on request from the corresponding author.
